# Characterization of the complete chloroplast genome of *Alopecurus pratensis* L. (Poaceae) 

**DOI:** 10.1080/23802359.2021.1935346

**Published:** 2021-07-14

**Authors:** Junfeng Yang, Wenxuan Du, Yongzhen Pang

**Affiliations:** Institute of Animal Sciences, Chinese Academy of Agricultural Sciences, Beijing, China

**Keywords:** *Alopecurus pratensis* L., chloroplast genome, phylogenetic relationship

## Abstract

*Alopecurus pratensis* L. is one of the most important fodder grasses distributed in sub-frigid regions of the world. In this study, the complete chloroplast genome of *A. pratensis* was deciphered and is 136,157 bp in length. The genome includes a large single-copy region of 80,275 bp, small single-copy region of 12,830 bp, and a pair of inverted repeat regions of 21,526 bp. The GC content of the complete chloroplast genome of *A. pratensis* is 38.30%. Among the 134 unique genes in the circular genome, 38 tRNA, eight rRNA, and 88 protein-coding genes were annotated. We constructed the Maximum likelihood (ML) tree with 13 species from the Poaceae and found that *A. pratensis* was phylogenetically related to *A. arundinaceus*. The published *A. pratensis* chloroplast genome will provide useful information for phylogenetic and evolutionary study of the genus *Alopecurus* in the Poaceae.

*Alopecurus pratensis* L. is a rhizomatous grass indigenous to the temperate parts of Europe and Asia (Hannaway and McGuire 1981). It is well-adapted to cool, moist environments, and it tolerates drought condition (Hannaway and McGuire 1981; Sheley 2007). *Alopecurus pratensis* is an early-growing cool-season grass that can grow throughout the winter in warmer climates. It can withstand flooding and survive in alkaline wetlands with a soil pH of up to 8.5 (Schoth 1945). Moreover, *A. pratensis* has a higher forage yield (e.g., tall plants and abundant leaves) and quality (e.g., softer stems and leaves, and better palatability) (Schoth 1945; Wenick et al. 2008). In this study, we sequenced and assembled the complete chloroplast genome of *A. pratensis* in order to provide genomic and genetic resources for further investigations.

Seeds of *A. pratensis* were originally acquired from Federal Research Center of Russia Vavilov Institute of Plant Genetic Resources (VIR) and stored at the Forage Germplasm Bank at Institute of Animal Sciences of the Chinese Academy of Agricultural Sciences (Beijing, E116°29′, N40°03′). The voucher specimen (FG2435) was deposited at the Herbarium of the Institute of Animal Sciences of the Chinese Academy of Agricultural Sciences, Beijing, China (http://ias.caas.cn/, Yongzhen Pang, pangyongzhen@caas.cn). After germination in the lab, genomic DNA from the young leaves was extracted using a DNA Extraction Kit from Tiangen Bio Tech Co., Ltd (Beijing, China). The sequencing was carried out on the Illumina Novaseq PE150 platform (Illumina Inc, San Diego), and 150 bp paired-end reads were generated. The software GetOrganelle v1.5 (Jin et al. 2018) was used to assemble the cleaned reads into a complete chloroplast genome. The chloroplast genome annotation was performed through the online program CPGAVAS2 (Shi et al. 2019) and GeSeq (Tillich et al. 2017), followed by manual correction. The assembled chloroplast genome sequence was then submitted to GenBank under the accession number MW309817.

The chloroplast genome of *A. pratensis* is 136,157 bp in length and contains a large single-copy region (LSC) of 80,275 bp, a small single-copy region (SSC) of 12,830 bp, and a pair of inverted repeat (IR) regions of 21,526 bp. The genome annotation predicted 134 genes, including 88 protein-coding, 38 tRNA, and eight rRNA genes. Nineteen genes contain introns, 18 (10 protein-coding and eight tRNA genes) of which contain one intron and one of which (ycf3) contains two introns. The overall GC content of the chloroplast genome is 38.30%, with the corresponding values in the LSC, SSC, and IR regions are 39.68%, 35.92% and 36.43%, respectively.

The chloroplast genomes of 13 plant species from the Poaceae were downloaded from the NCBI GenBank database to identify the phylogenetic relationship of *A. pratensis*. The sequences were aligned using default settings in MAFFT v7 (Katoh et al. 2019). In addition, a Maximum likelihood (ML) tree based on the common protein-coding genes of all 14 species was constructed using RAxMLGUI1.5b (v8.2.12) (Silvestro and Michalak 2012) with nucleotide substitution model (HIVb + F+I) and 1000 bootstrap replicates. Phylogenetic analysis suggested that *A. pratensis* is closely clustered with *A. arundinaceus*, *A. aequalis* and *A. japonicus* (Figure 1). This study indicated that the plants of the genera of *Poa* and *Alopecurus* are evolutionarily closely related, but the species within each genus are clustered together, which is similar to previous studies (Bouchenak-Khelladi et al. 2008; Orton et al. 2019). This research lays a foundation for further investigations and the possible bioengineering of the chloroplast genome of *A. pratensis*.

**Figure 1.**
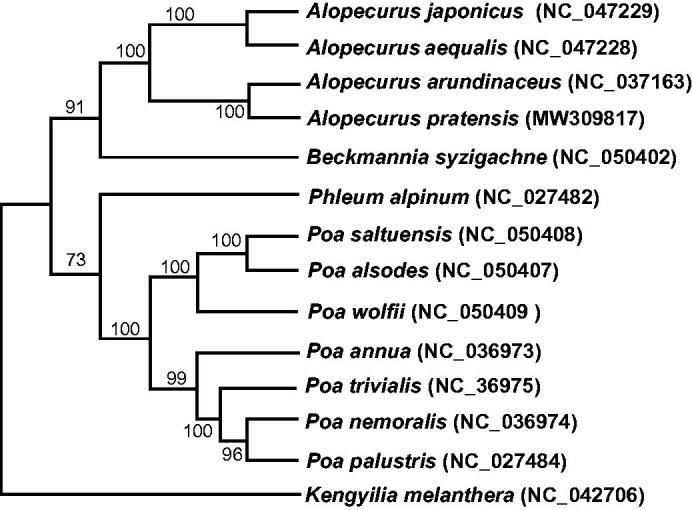
Maximum likelihood phylogenetic tree of *Alopecurus pratensis* based on 14 complete chloroplast genome sequences using *Kengyilia melanthera* as the outgroup. Numbers in the nodes are bootstrap values based on 1000 replicates and the best model chosen was HIVb + F+I.

## Data Availability

The genome sequence data that support the findings of this study are openly available in GenBank of NCBI at [https://www.ncbi.nlm.nih.gov] under the accession no. MW309817. The associated BioProject, SRA, and Bio-Sample numbers are PRJNA689884, SRP301016, and SAMN17221515, respectively.
